# Bovine Mastitis and Detection of *Staphylococcus aureus* Among Dairy Cows in Dessie Town, Northeast Ethiopia: Prevalence and Associated Risk Factors

**DOI:** 10.1155/vmi/9069263

**Published:** 2026-10-08

**Authors:** Sossina Ashenafi Bijiju, Habtamu Tassew Tarekegne, Halo Yohannes Alemu, Solomon Tibebu Melkie

**Affiliations:** ^1^ Department of Veterinary Biomedical and Para-Clinical Sciences School of Veterinary Medicine Bahir Dar University P.O.Box: 5501 Bahir Dar Ethiopia bdu.edu.et

**Keywords:** bovine mastitis, dairy cattle, Ethiopia, prevalence, risk factors, *Staphylococcus aureus*, subclinical mastitis

## Abstract

**Background:**

Bovine mastitis is a major economic challenge to dairy production globally. Despite its impact, there is limited information on the disease and its associated risk factors in the dairy sector of Dessie Town, Northeast Ethiopia. This study aimed to determine the prevalence of bovine mastitis, identify risk factors, and isolate *Staphylococcus aureus* from mastitic milk in the study area.

**Methods:**

A cross‐sectional study was conducted on 304 lactating dairy cows across smallholder farms and semi‐intensive farms. Clinical examination and the California mastitis test (CMT) were used to detect clinical and subclinical mastitis, respectively. Risk factors were assessed via structured questionnaires and analyzed through univariate and multivariable logistic regression. Out of 374 mastitis‐positive quarters identified, 52 were blind teats; consequently, a bacteriological analysis was performed on the remaining 322 viable milk samples for the exclusive isolation of *Staphylococcus aureus* via standard microbiological techniques.

**Results:**

The cow‐level prevalence of mastitis was 61.18%, with the prevalence of subclinical cases (48.68%) far exceeding that of clinical cases (12.5%). At the quarter level, 30.75% of the teats were affected. The right rear quarter showed the highest prevalence (34.21%), whereas the left front quarter was least affected (27.63%). Multivariable logistic regression revealed that crossbreed (AOR = 10.15; 95% CI: 2.52–40.94; *p* = 0.001), many parity (AOR = 20.05; 95% CI: 3.32–147.24; *p* = 0.001), poor milking hygiene (AOR = 5.64; 95% CI: 1.35–23.52; *p* = 0.017), and long and wide teat end morphology (AOR = 9.03; 95% CI: 2.15–37.98; *p* = 0.003) were significant predictors of mastitis occurrence. *Staphylococcus aureus* was isolated from 35.71% of mastitis‐positive milk samples, including 37.36% from clinical samples and 35.06% from subclinical samples.

**Conclusion:**

This study revealed a high burden of both clinical and subclinical mastitis in dairy farms around Dessie Town, with *S. aureus* being a leading pathogen. The strong association between mastitis and risk factors such as hygiene and teat anatomy highlights the need for integrated control measures, farmer training, and routine screening to improve udder health and milk quality.

## 1. Background

Ethiopia has the largest livestock population in Africa, with cattle playing a central role in the livelihoods and food security of rural communities [1]. Among the national cattle herd, approximately 55.90% are female, with approximately 20.7% being lactating cows, which are primarily composed of indigenous breeds, and a smaller proportion of crossbreeds and exotics introduced to improve dairy productivity [2]. While the dairy sector is gradually transitioning from traditional extensive systems to semi‐intensive and intensive production in peri‐urban areas, infectious diseases remain a major constraint on productivity, milk quality, and animal health [3, 4].

Bovine mastitis is one of the most prevalent and economically important diseases affecting dairy cows globally, with significant impacts on milk yield, quality, animal welfare, and public health [5]. It is characterized by inflammation of the mammary gland parenchyma and exists in clinical and subclinical forms [6, 7]. Clinical mastitis is easily detectable due to visible symptoms such as swelling, redness, pain, and abnormalities in milk, including clotting or discoloration [8]. In contrast, subclinical mastitis presents no visible signs on the udder or in the milk, making it more challenging to diagnose. Despite the absence of obvious symptoms, subclinical mastitis can significantly impact milk production and quality, often requiring laboratory tests for detection. Depending on the mode of transmission, mastitis can be classified as environmental, linked to pathogens present in the cow’s surroundings, or contagious, where the infection spreads primarily during milking from infected udders [9].

Among the numerous pathogens associated with contagious mastitis, *Staphylococcus aureus* is one of the most frequently isolated bacteria. It is a Gram‐positive, coagulase‐positive, opportunistic pathogen that persists in the mammary gland, evades host immune responses, and leads to chronic and recurrent infections [10, 11]. Its ability to form biofilms and develop resistance to antimicrobials poses a dual threat to animal health and public health. Infected milk, especially in regions where raw milk consumption is common, serves as a reservoir for zoonotic transmission and foodborne illness [12, 13]. In Ethiopia, several studies have reported *S. aureus* as a major etiological agent in bovine mastitis, which is often isolated in both clinical and subclinical cases [14–16].

The economic consequences of *S. aureus*‐associated mastitis are considerable. These include milk losses, treatment costs, premature culling of affected animals, reduced milk shelf‐life, and drug residues in milk [5, 17]. In Ethiopian crossbreds, quarters with subclinical *S. aureus* lost 34.5% of potential milk production, with a total milk yield loss of 6.8% per cow per lactation, and the overall financial loss for each cow per lactation was estimated to be US$150.35 for large farms [18]. Furthermore, the spread of antimicrobial‐resistant strains of *S. aureus* exacerbates concerns regarding food safety and effective treatment options, aligning with the broader challenges addressed through alternatives such as herbal medicine [19, 20]. Despite increasing recognition of this issue, region‐specific data are lacking in urban and peri‐urban dairy farms in Ethiopia, including Dessie town. In these areas, rapid urbanization and a growing consumer population have created a significant demand‐driven potential for dairy expansion [21, 22].

Although some studies have examined mastitis prevalence and etiology in various parts of Ethiopia, limited recent evidence exists from Dessie Town, Amhara Region. This data gap is concerning given the rapid intensification of dairy farming in the region, which often occurs without improvements in hygienic measures. Without localized data on the prevalence of both clinical and subclinical mastitis and the specific role of *S. aureus*, it is challenging to design evidence‐based control strategies or antimicrobial stewardship programs tailored to these unique regional conditions. Most Ethiopian mastitis studies use cross‐sectional clinical/California mastitis test (CMT) screening, questionnaire‐based risk‐factor data with multivariable regression, and standard bacteriological culture and biochemical (sometimes molecular) methods to isolate and identify *Staphylococcus aureus* and other pathogens from aseptic milk samples [23–25]. Therefore, the aim of this study was to determine the prevalence of bovine mastitis, identify associated risk factors, and isolate *Staphylococcus aureus* from mastitic milk in lactating dairy cows in Dessie Town, Northeast Ethiopia.

## 2. Methods

### 2.1. Description of the Study Area

The study was conducted in Dessie town, which is located in the South Wollo Zone of the Amhara Region, Northeast Ethiopia. Dessie lies approximately 401 km north of Addis Ababa at an elevation ranging from 2470 to 2550 m above sea level. The town is situated at 11°8′N latitude and 39°38′E longitude [26], with an average annual air temperature of about 17°C–18°C [27, 28].

### 2.2. Study Population and Husbandry Practices

A total of 304 lactating dairy cows, comprising both local and crossbred (Holstein Friesian × local zebu) animals, were selected from 28 dairy farms employing either intensive or semi‐intensive management systems. Cows were included if they were actively lactating crossbred or local breeds in any parity or lactation stage; cows in their dry period, aggressive animals, or those under active antibiotic treatment within the prior 2 weeks were excluded. Cows in intensive systems were housed indoors and provided with concentrated feed, hay, green forage, and crop residues. Semi‐intensively managed cows were allowed to graze during the day and were supplemented with feed during morning and evening milking sessions. These classifications follow standard criteria based on grazing access and degree of stall‐feeding [29–31]. Cows were categorized on the basis of age (young: < 4 years, adult: 4–8 years, old: > 8 years), parity (few: < 3 calvings, moderate: 3–6 calvings, many: ≥ 7 calvings), lactation stage (early: ≤ 121 days, middle: 121–240 days, late: > 240 days), and milk yield (low: < 4 L/day, medium: 4–9 L/day, high: > 9 L/day). Teat end morphology and body condition scores were also recorded via standard classification systems reported in prior studies [32, 33]. Milk samples were collected from dairy cows maintained in intensive and semi‐intensive dairy farms located in Dessie Town, Northeastern Ethiopia. The dairy cows used in this study were not purchased; they were sourced directly from farm owners who rear their own cattle in the study area. All the animals used were under normal farm management with no experimental treatment or intervention.

### 2.3. Study Design and Sampling Techniques

A cross‐sectional study was conducted from December 2023 to July 2024 to estimate the prevalence of bovine mastitis, identify associated risk factors, and isolate *Staphylococcus aureus* from mastitic milk. The sample size was calculated via the formula described by Thrusfield [34].

(1) n=Z2∗Pexp1−Pexpd2,

where *Z* = 1.96: For a 95% confidence interval (CI); *P*exp = 27.2% (assuming previous prevalence by [35]); *d *= 0.05: Desired absolute precision (5% margin of error). A multistage sampling design was used. A total of 28 dairy farms were selected via convenience sampling (based on their accessibility, the presence of at least one lactating cow, and the willingness of owners to participate), and 304 lactating cows were then randomly chosen via the lottery method. 304 lactating cows were proportionally distributed among 28 farms by simple random sampling.

### 2.4. Physical Examination and Milk Sample Collection

Each udder was visually inspected and palpated to detect clinical signs such as swelling, heat, fibrosis, or atrophy. Milk from each quarter was examined for abnormalities, including blood, clots, or flakes. For subclinical mastitis, the CMT was used. Before sampling, the teats were thoroughly cleaned with water and dried with individual disposable towels to prevent cross‐contamination. To ensure the milk samples were collected aseptically and originated solely from the intramammary environment, each teat end was scrubbed with cotton wool moistened with 70% ethyl alcohol. The disinfection process followed a strict sequence, starting with the teats furthest from the collector and ending with the nearest teats, while sampling was performed in the reverse order. The first 3–4 streams of milk were discarded to remove any bacteria present in the teat canal. Approximately 10 mL of milk was then collected into sterile, prelabeled universal bottles held nearly horizontally to prevent the entry of skin flora or environmental dust into the containers [36]. The samples were stored in iceboxes and transported to the Veterinary Microbiology Laboratory at Wollo University. The clinical samples were cultured immediately, while the other samples were stored at 4°C for a maximum of 24 h prior to processing.

### 2.5. CMT

Subclinical mastitis was detected via the CMT as described by Moawad et al. [37]. Equal volumes of milk and CMT reagent were mixed in the CMT paddle, and the degree of gel formation was scored as 0 (negative), trace, + (weak), ++ (distinct), or +++ (strong). Quarters scoring + or higher were considered positive. Cows were classified as subclinically infected if at least one‐quarter tested positive [37].

### 2.6. Isolation and Identification of *Staphylococcus aureus*

Isolation of *S. aureus* was performed following the procedures described by Zang et al. [38]. Briefly, 10 μL (0.01 mL) of milk was streaked onto 5% sheep blood agar and incubated aerobically at 37°C for 24–48 h. Colonies were evaluated for morphology and hemolysis and then subcultured on nutrient agar for further purification. Gram‐positive cocci showing catalase‐positive reactions were tested via slide or tube coagulase tests. Coagulase‐positive colonies were further confirmed by growth on mannitol salt agar. *S. aureus* ATCC 25923 was utilized as a quality control strain to validate the microbiological procedures.

### 2.7. Questionnaire Survey

A semistructured questionnaire was administered to 65 respondents (comprising farm owners, managers, and active milkers) across the 28 farms to gather information on herd‐level and animal‐level factors. These included farm hygiene, milking practices, floor type, breed, age, parity, body condition score, lactation stage, teat morphology, milk yield, history of mastitis, concurrent illnesses, dry cow therapy, and udder/milk abnormalities. The questionnaire was pretested on 5 nonstudy dairy farms in the area to check for clarity and flow.

### 2.8. Data Analysis

All the data were entered into Microsoft Excel and analyzed via STATA Version 14 (StataCorp, College Station, TX, USA). Descriptive statistics were used to calculate the prevalence. Pearson’s chi‐square test was applied to examine associations between potential risk factors and mastitis occurrence. Variables with *p* < 0.2 in the univariate analysis were entered into the multivariable logistic regression to identify independent predictors. Backward elimination was performed, retaining variables with *p* < 0.05. Highly collinear variables (variance inflation factor [VIF] > 5), such as age, were excluded to ensure model stability. Odds ratios (ORs) and 95% CIs were reported, and significance was considered at *p* < 0.05. Model fitness was assessed using the Hosmer–Lemeshow goodness‐of‐fit test. Multicollinearity among the explanatory variables was evaluated using the VIF, with a VIF < 5 indicating the absence of significant collinearity.

### 2.9. Ethical Consideration

All the experimental procedures using animals were reviewed and cleared by the Research and Ethics Committee of the College of Agriculture and Environmental Sciences, Bahir Dar University (Ref No: 1/Vetsc/342/1‐1–3, dated 15 October 2024). All the procedures were conducted following applicable institutional, national, and international guidelines and regulations, including the OIE Manual for Sample Collection [39] and ARRIVE guidelines 2.0 [40]. Informed consent was obtained from all respondents.

## 3. Results

### 3.1. Prevalence of Bovine Mastitis

Among the 304 lactating dairy cows and 1216 quarters examined, the overall mastitis prevalence was 61.18% at the cow level and 30.75% at the quarter level. Subclinical mastitis was more prevalent than clinical mastitis at both the individual animal and quarter levels. These findings are summarized in Table 1.

**TABLE 1:** Prevalence of bovine mastitis.

Type of mastitis	Cow‐level prevalence % (*n*)	95% CI	Quarter‐level prevalence % (*n*)	95% CI
Clinical Mastitis	12.50 (38)	9.04–16.71	11.75 (143)	10.01–13.70
Subclinical Mastitis	48.68 (148)	42.94–54.46	18.99 (231)	16.83–21.32
Total	**61.18 (186)**	**55.45–66.69**	**30.75 (374)**	**28.18–33.43**

### 3.2. Distribution of Mastitis Among Teat Quarters

Mastitis susceptibility varied across the four anatomical quarters, with the highest overall prevalence recorded in the right rear (RR) quarter (34.21%, *n* = 104) and the lowest in the left front (LF) quarter (27.63%, *n* = 84). Blind teats accounted for an overall loss of 4.27% (*n* = 52) of the total quarters examined, occurring most frequently in the hindquarters. The detailed quarter‐level data are presented in Table 2.

**TABLE 2:** Prevalence of subclinical and clinical mastitis at the quarter level.

Quarter	No. of teats examined	Overall mastitis % (*n*)	Subclinical mastitis % (*n*)	Clinical mastitis % (*n*)	Blind teat % (*n*)
RF	304	28.94 (88)	21.71 (66)	7.2 (22)	2.6 (8)
RR	304	34.21 (104)	19.40 (59)	14.8 (45)	3.95 (12)
LR	304	32.23 (98)	19.07 (58)	13.15 (40)	5.59 (17)
LF	304	27.63 (84)	15.78 (48)	11.84 (36)	4.93 (15)
Total	**1216**	**30.75 (374)**	**18.99 (231)**	**11.75 (143)**	**4.27 (52)**

### 3.3. Potential Factors Associated With Bovine Mastitis

Descriptive analysis of potential demographic and management‐related risk factors revealed varying mastitis prevalence across categories. Older cows (> 8 years), crossbreeds, poor body condition scores, many parity (≥7 calvings), late‐stage lactation (> 240 days), poor milking and farm hygiene, and soil flooring were associated with markedly high mastitis prevalence. Cows with short and narrow teat ends, no dry cow therapy, and a history of previous mastitis also demonstrated elevated mastitis occurrence. The descriptive distribution of mastitis across these variables is presented in Table 3.

**TABLE 3:** Potential factors associated with the occurrence of bovine mastitis.

Variables	Category	No. Examined	Prevalence of mastitis % (*n*)	95% CI
Age	Young (< 4 years)	75	17.33 (13)	9.56–27.82
	Adult (4–8 years)	103	32.02 (33)	23.16–42.01
	Old (> 8 years)	126	77.77 (98)	69.57–84.66

Breed	Local	133	16.54 (22)	10.66–23.97
	Crossbreed	171	71.92 (123)	64.55–78.47

Body condition score	Good	133	36.84 (49)	28.64–45.69
	Medium	146	52.05 (76)	43.62–60.41
	Bad	25	84.0 (21)	63.92–95.46

Parity	Few (< 3 calves)	104	7.69 (8)	3.37–14.61
	Moderate (3–6 calves)	92	34.78 (32)	25.17–45.36
	Many (≥ 7 calvings)	108	96.29 (104)	90.79–98.92

Lactation stage	Early (< 121 days)	119	3.36 (4)	0.92–8.37
	Middle (121–240 days)	73	49.3 (36)	37.42–61.27
	Late (> 240 days)	112	91.96 (103)	85.32–96.16

Farm hygiene	Good	213	42.72 (91)	35.99–49.67
	Bad	91	58.24 (53)	47.41–68.51

Milking hygiene	Good	136	9.55 (13)	5.19–15.78
	Poor	168	77.97 (131)	70.93–83.99

Milk yield	Low (< 4 Lt)	153	20.26 (31)	14.21–27.53
	Medium (4–9 Lt)	140	62.1 (87)	53.56–70.21
	High (> 9 Lt)	11	18.18 (2)	2.28–51.78

Floor type	Concrete	200	26.5 (53)	20.54–33.22
	Soil	104	82.69 (86)	74.01–89.43

Teat end morphology	Short and narrow	171	11.11 (19)	6.83–16.81
	Long and wide	133	90.22 (120)	83.89–94.67

Dry cow therapy	Yes	129	37.98 (49)	29.58–46.96
	No	125	60.8 (76)	51.68–69.37

Concurrent diseases	Yes	180	20.55 (37)	14.91–27.23
	No	124	85.48 (106)	78.02–91.21

Previous mastitis history	Yes	143	72.72 (104)	64.67–79.83
	No	201	24.87 (50)	19.06–31.46

*Note:* The relatively wide 95% confidence intervals observed for specific subcategories are due to small sample size.

Abbreviation: CI, confidence interval.

### 3.4. Univariable Logistic Regression Analysis

Univariate logistic regression identified several factors significantly associated with mastitis (*p* < 0.05). These included age, breed, body condition score, parity, lactation stage, teat end morphology, and previous mastitis history. For example, older cows (> 8 years) were more likely to have mastitis than younger cows were (OR = 15.68; 95% CI: 8.24–29.81). Similarly, crossbred cows had significantly greater odds of mastitis than local breeds did (OR = 68.57; 95% CI: 35.35–132.98). The results of the univariable analysis are shown in Table 4.

**TABLE 4:** Univariable logistic regression analysis of potential risk factors associated with mastitis.

Variables	Category	Prevalence (%)	OR	95% CI	(*p* value)
Age	Young	17.33	Ref	Ref	Ref
	Adult	32.02	2.18	1.15–4.16	0.016
	Old	77.77	15.68	8.24–29.81	0.001

Breed	Local	16.54	Ref	Ref	
	Crossbreed	71.92	68.57	35.35–132.98	0.001

Body condition score	Good	36.42	Ref	Ref	Ref
	Medium	52.84	1.92	1.26–2.93	0.002
	Bad	84.0	9.60	3.16–29.12	0.001

Parity	Few	7.69	Ref	Ref	Ref
	Moderate	34.78	6.37	3.01–13.48	0.001
	Many	96.26	319.44	106.17–961.08	0.001

Lactation stage	Early	3.36	Ref	Ref	
	Middle	49.3	22.98	9.22–57.27	0.001
	Late	91.96	285.81	102.81–794.59	0.001

Farm hygiene	Good	42.72	Ref	Ref	
	Bad	58.24	26.94	15.33–47.35	0.001

Milking hygiene	Good	9.55	Ref	Ref	
	Poor	77.97	35.18	19.13–64.72	0.001

Milk yield	< 4 L	20.26	Ref	Ref	0.001
	4–9 L	62.1	6.45	3.82–10.89	
	Above 9 L	18.18	0.87	0.17–4.39	

Floor type	Yes	26.5	Ref	Ref	
	No	82.69	11.36	6.72–19.21	0.001

Teat end morphology	Short and narrow	11.11	Ref	Ref	
	Long and wide	90.22	126.4	58.69–272.22	0.001

Dry cow therapy	Yes	37.98	Ref	Ref	Ref
	No	60.8	2.52	1.66–3.83	0.001

Concurrent disease	Yes	20.55	Ref	Ref	Ref
	No	85.48	23.50	13.5–40.87	0.001

Previous mastitis history	Yes	72.72	Ref	Ref	Ref
	No	24.87	0.124682	0.079–0.19	0.001

*Note:* ^∗^ *p* < 0.05 (significant).

Abbreviation: OR, odds ratio.

### 3.5. Multivariable Logistic Regression Analysis

Multicollinearity assessment showed a high correlation between age and parity (VIF > 5); consequently, age was excluded from the final model to maintain stability. All other remaining variables demonstrated acceptable VIF < 2.0. In the multivariable model, four variables remained significantly associated with mastitis. Compared with local breeds, crossbred cows were 10.15 times more likely to develop mastitis (95% CI: 2.52–40.94; *p* = 0.001). Higher parity and poor milking hygiene are also strongly correlated, with cows having ≥7 calvings being 20.05 times more likely to have mastitis than cows with fewer calvings (95% CI: 3.32–147.24; *p* = 0.001). Teat end morphology was a significant predictor, with cows having long and wide teats showing significantly increased odds (AOR = 9.03; 95% CI: 2.15–37.98; *p* = 0.003). The full results are presented in Table 5.

**TABLE 5:** Multivariable logistic regression analysis of potential risk factors associated with mastitis.

Variables	Category	Prevalence (%)	AOR	95% CI	(*p* value)
Breed	Local	16.56	Ref	Ref	
	Crossbreed	71.62	10.15	2.52–40.94	0.001

Parity	Few	7.63	Ref	Ref	
	Moderate	34.5	4.77	1.81–12.53	0.002
	Many	96.35	20.05	3.32–147.24	0.001

Milking hygiene	Good	9.30	Ref	Ref	
	Poor	78.3	5.64	1.35–23.52	0.017

Teat end morphology	Short and narrow	11.11	Ref	Ref	
	Long and wide	90.47	9.03	2.15–37.98	0.003

*Note:* *p* < 0.05 (significant).

Abbreviation: AOR, adjusted odds ratio.

### 3.6. Occurrence of *Staphylococcus aureus* in Mastitic Milk

Out of the 374 affected quarters, milk samples were successfully collected from only 322 quarters for bacterial culture; the remaining 52 quarters were blind teats from which no milk could be obtained. Among the 322 milk samples analyzed from mastitic cows, *S. aureus* was isolated from 115 samples (35.71%). Among these isolates, 34 (37.36%) were recovered from clinical mastitis cases, whereas 81 (35.06%) were from subclinical cases. These findings indicate the widespread occurrence of *S. aureus* across both clinical and subclinical mastitis patients. The isolation frequencies are summarized in Table 6.

**TABLE 6:** *Staphylococcus aureus* isolates.

Mastitis type	Milk samples	No. of positive	Prevalence (%)
Clinical mastitis	91	34	37.36
Subclinical mastitis	231	81	35.06
Total	322	115	35.71

## 4. Discussion

The present study revealed a considerable cow‐level prevalence of bovine mastitis at 61.18% (*n* = 186), with subclinical mastitis (48.68%) being the dominant form, occurring nearly four times more frequently than clinical mastitis (12.5%). These findings align with previous reports from Ethiopia, such as those by Lakew et al. [6] in Haramaya (63.02%) and Abebe et al. [23] in Hawassa (62.6%), suggesting a consistent burden across different regions. The comparable prevalence rates may be attributed to shared risk factors, including inadequate milking hygiene, suboptimal housing conditions, and the absence of routine mastitis screening programs. However, the current overall prevalence is lower than the 80% prevalence reported by Mbindyo et al. [41] in Kenya. Conversely, the prevalence reported in this study exceeds that reported by Dabele et al. [42] in the West Shewa Zone (30.5%) and Belay et al. [25] in the Gamo Zone (17.1%). These variations underscore the influence of diverse environmental conditions, breed differences, and management practices on the epidemiology of mastitis.

The prevalence of cow‐level clinical mastitis (12.5%) recorded in this study is consistent with the 12.5% prevalence reported by Zeryehun and Abera [43] in East Hararghe. However, these findings are lower than those of Fesseha et al. [44] in Modjo Town (28.9%) and Mekibib et al. [24] in Holeta (22.4%), where clinical cases were more frequently observed. Variation might be due to differences in mastitis detection methods, farmer awareness, and veterinary intervention strategies. The relatively high clinical mastitis burden in this study could be linked to the absence of dry cow therapy, delayed treatment of subclinical infections, and the presence of concurrent diseases that compromise udder immunity.

Subclinical mastitis remains a more significant challenge, with a prevalence of 48.68%, which is in agreement with the findings of Mekibib et al. [24] in Holeta (48.6%). However, it is lower than the findings of Shiferaw and Telila [45] (61.19%) in Wolayta Sodo, Zeryehun and Abera [43] (51.8%) in East Hararghe, and Fesseha et al. [44] (71.02%) in Modjo Town. The observed variations could stem from differences in sampling periods, diagnostic approaches, and levels of farmer awareness regarding subclinical mastitis. Historically, the detection of subclinical mastitis has been challenging, as its asymptomatic nature leads to underestimation by farmers and delays in intervention. Over time, increased awareness and advancements in diagnostic methods, such as the CMT and somatic cell count (SCC) techniques, have facilitated better detection and reporting. In contrast, the current prevalence of subclinical mastitis is higher than that reported by Kebebew and Jorga [46] in Ambo (31.6%) and Demissie et al. [47] in Wukro Tigray (26%). This discrepancy may be explained by variations in climatic conditions, parity, lactation stage, breed susceptibility, and farm management practices. Notably, Erskine [48] emphasized that subclinical mastitis consistently surpasses clinical mastitis because the udder’s protective immune responses limit severe clinical manifestations. The higher prevalence of subclinical mastitis compared to clinical mastitis is attributed to its hidden nature of the infection; it causes no visible changes to the milk or udder, allowing it to go unnoticed during routine manual milking. This pattern reflects a widespread lack of regular farm screening, such as the CMT. Without early detection, these silent infections remain untreated, creating a permanent reservoir for pathogens like *S. aureus* to spread through the herd while quietly draining milk yields.

The overall quarter‐level prevalence of mastitis in this study was 30.75%, which is higher than the 26.9% reported by Tezera and Ali [49] in Assosa town and the 21.94% reported by Shiferaw and Telila [45] in Wolaita. Similarly, lower prevalence rates were reported by Belay et al. [25] (7.94%) and Demissie et al. [47] (15.19%) elsewhere in Ethiopia. The disparity in prevalence rates may be attributed to variations in farm management, udder hygiene, and environmental sanitation. Poor hygiene practices, such as inadequate cleaning and drying of cow housing and udder surfaces, could have contributed to the higher prevalence observed in the present study. Conversely, our findings were lower than the 36.9% reported by Fesseha et al. [44], which could be explained by differences in climatic conditions and farm biosecurity measures.

In this study, the quarter‐level prevalence of clinical mastitis was 11.75%, which closely aligns with the 11.9% reported by Tezera and Ali [49] in Assosa and the 13.3% documented by Demissie et al. [47] in Wukro, Tigray. However, this prevalence was higher than the 5.2% reported by Zeryehun et al. [50] and the 3.4% recorded by Musse et al. [51] in Addis Ababa, which may reflect differences in dairy management systems, climates, and milking hygiene. Extreme environmental conditions—either excessively hot or cold—can also influence the survival and proliferation of mastitis‐causing pathogens, potentially impacting the variation in prevalence across different regions.

Subclinical mastitis at the quarter level was identified in 18.99% of the examined teats, which is comparable to the 17.9% reported by Almaw et al. [52]. However, this prevalence was lower than those reported by Fesseha et al. [44] (34.9%) and Zeryehun and Abera [43] (46.4%), but higher than the 2.6% reported in Wukro, Tigray [47]. Differences in detection methods, study durations, sample sizes, and farm management practices may explain these inconsistencies. Subclinical mastitis remains a major concern, as it often goes undetected, leading to chronic infections, reduced milk production, and economic losses.

Among the four quarters, the RR teat presented the highest infection rate (34.21%), followed by the left rear (32.23%), which aligns with several studies [36, 53]. The higher susceptibility of the rear quarters to infection may be attributed to their greater milk production capacity and increased exposure to environmental contaminants, particularly fecal matter [8]. Additionally, this study revealed that 4.3% of the teats were blind, which is consistent with previous reports by Fesseha et al. [44] (3.4%) and Kebebew & Jorga [46] (5.5%) but lower than the prevalence reported by Biffa et al. [54]. The presence of blind quarters may indicate chronic mastitis infections, inadequate treatment of clinical cases, and the absence of effective culling practices. The failure to identify and treat subclinical mastitis early may contribute to the progression of infections, leading to irreversible damage to the mammary gland. This, in turn, reduces milk yield, impacts dairy productivity, and poses food security concerns in the region.

This study investigated the associations between various demographic and management‐related risk factors and the prevalence of bovine mastitis. The findings revealed significant associations between mastitis occurrence and factors such as breed, parity, milking hygiene, teat end morphology, and previous mastitis history, which aligns with the literature on the subject.

A statistically significant correlation was observed between breed type and mastitis prevalence, with crossbred cows being more susceptible than local breeds. Compared with local breeds, crossbred breeds had a 10.15‐fold greater likelihood of developing mastitis, which is consistent with previous reports [32, 33]. The increased susceptibility of crossbred cows may be attributed to their greater milk production capacity, larger udder size, and reduced genetic resistance to infections. Local breeds, on the other hand, have developed a natural resilience to mastitis due to their adaptation to the environment and lower milk yield, which reduces stress on the udder and decreases the risk of microbial invasion.

This study revealed a strong statistical relationship between parity and mastitis occurrence. Cows with moderate parity (3‐6 calvings) were 4.77 times more likely to develop mastitis than those with few calvings (<3), whereas cows with many calvings (≥7) presented a similar increase in risk. This pattern supports findings from a previous study [25], which indicated that repeated lactation cycles contribute to increased susceptibility. The heightened risk in multiparous cows could be due to anatomical and physiological changes in the udder, cumulative tissue stress, and prolonged exposure to environmental and infectious agents.

Milking hygiene practices were significantly associated with mastitis prevalence, with cows subjected to poor milking hygiene being 5.64 times more likely to develop mastitis. These findings are consistent with those of previous studies [24, 50]. Poor hygiene practices, such as inadequate udder washing, the use of a common cloth for multiple cows, and milking of subclinically mastitic cows first, have been reported as major risk factors for contagious mastitis. Proper hygiene practices, including individual udder cloths and pre‐ and postmilking teat disinfection, are critical control measures to mitigate the spread of mastitis‐causing pathogens.

Teat end morphology was another significant risk factor associated with mastitis incidence. Cows with long and wide teat ends were 9.03 times more likely to develop mastitis than those with short and narrow teat ends. This result is in agreement with findings from [23, 55], who reported an increased risk of mastitis in cows with wide or flat teat‐end shapes. The greater susceptibility may be due to the wider streak canals in such teats, which facilitate pathogen entry and increase bacterial colonization, ultimately leading to mastitis. These findings point to the need for practical training of dairy farmers on proper milking hygiene and selective breeding for anatomical traits that are less prone to mastitis. It is important to note that the relationship between teat end morphology and mastitis may involve reverse causality. While poor teat end morphology, such as hyperkeratosis, serves as a primary risk factor by facilitating bacterial colonization, it may also be a consequence of chronic *S. aureus* infection. The persistent immune response and leukocyte infiltration associated with chronic mastitis can lead to tissue remodeling, fibrosis, and a loss of sphincter elasticity, thereby worsening the physical appearance of the teat end [56]. The exceptionally high univariable ORs observed for high parity and late lactation stage come from very low baseline prevalence in their respective reference groups (early lactation and few calvings). Biologically, this reflects the cumulative exposure of udders to pathogens over multiple years in semi‐intensive management systems lacking structured mastitis control programs. In the multivariable analysis, the wide 95% CI for high parity is due to smaller sub‐sample sizes in the highest‐risk group. This reduces precision but does not alter the strong biological significance of the risk factor.

The microbiological analysis conducted in this study revealed *Staphylococcus aureus* in 35.71% (115/322) of the mastitic milk samples. This finding is consistent with previous reports, such as those by Fesseha et al. [44] (40.3%) and Belay et al. [25] (42.6%). However, the prevalence reported by Asmelash et al. [57] (73.3%) in Kombolcha and Abebe et al. [23] (73.2%) was considerably higher than that reported in the present study. A lower percentage (13.3%) was reported in Gondar by Tegegne and Tesfay [58]. This variation may be attributed to differences in climatic conditions, which can influence bacterial survival and transmission. *S. aureus* has been widely reported as a primary etiological agent of bovine mastitis in multiple studies across Africa and Asia [55].

In the present study, the prevalence of *S. aureus* was 37.36% in clinical mastitis cases and 35.06% in subclinical mastitis cases, which is in agreement with the rates reported by Abera et al. [59] (33.3% and 44.5%, respectively). The higher *S. aureus* isolation in clinical mastitis compared to subclinical cases reflects the pathogen’s robust tissue damaging virulence factors that rapidly drive infections into acute inflammatory flare‐ups, a trend consistent with findings of [60]. The significant occurrence of *S. aureus* in this study may be associated with several management factors, including the absence of postmilking teat disinfection, the failure to cull chronically infected cows, the lack of dry cow therapy, and the common practice of hand milking. *S. aureus* is a highly contagious pathogen that colonizes the udder and teat surfaces of infected cows, serving as a key vector for transmission between infected and uninfected quarters, particularly during milking. Notably, although all the observed herds followed hand‐washing practices before milking, this was only performed before milking the first cow, which may have contributed to cross‐contamination during subsequent milkings.

## 5. Limitations of the Study

This study has several limitations that should be acknowledged. Firstly, the sample size was limited to a specific geographic area, which may restrict the generalizability of the findings to other regions with different management practices and climatic conditions. Secondly, the use of convenience sampling due to the absence of a complete dairy registry is a study limitation that may restrict the generalizability of these findings to the broader regional population. Thirdly, the study relied primarily on conventional microbiological methods for the isolation of *S. aureus* without molecular confirmation, which could affect the accuracy of species identification. Other mastitis‐causing pathogens were not investigated because of time and funding constraints. Fourthly, due to the cross‐sectional nature of this study, it is difficult to definitively establish the direction of the association between teat end morphology and mastitis. Poor morphology could be a predisposing factor for infection or a pathological consequence of long‐term intramammary inflammation caused by *S. aureus*. Additionally, while the questionnaire was pretested locally for clarity, it did not undergo a formal statistical validation process.

Furthermore, antimicrobial susceptibility testing (AST) and the genetic diversity of *S. aureus* strains were not assessed in this study due to the financial and time constraints associated with the DVM thesis project. The current findings provide a critical baseline for future research into the antimicrobial resistance profiles of mastitis pathogens in Northeast Ethiopia.

## 6. Conclusion

This study revealed a high prevalence of bovine mastitis among lactating cows in Dessie Town, with subclinical cases predominating over clinical cases, highlighting the silent yet substantial burden of the disease. The distribution of mastitis across quarters, particularly the higher incidence in rear teats, and the significant associations with various animal‐ and management‐related risk factors underscore the multifactorial nature of the disease. Multivariable analysis further identified breed, parity, milking hygiene, and teat end morphology as critical predictors of mastitis occurrence. The association with teat morphology suggests a potential clinicopathological link; while it may serve as a risk factor, chronic exposure to *S. aureus* can also lead to acquired pathological changes such as fibrosis and hyperkeratosis of the teat end, causing a loss of its innate defensive function. The isolation of *S. aureus* as the predominant pathogen in both clinical and subclinical cases confirms its central role in the epidemiology of mastitis in the study area. To effectively reduce mastitis in the study area, tailored interventions are required for key stakeholders: Farmers must prioritize routine postmilking teat dipping, strict milking hygiene, and maintaining teat tissue health; veterinarians should implement early screening protocols for subclinical mastitis and deliver practical extension education on herd demographics and management; and policymakers need to establish structured national mastitis control strategies and educational frameworks to safeguard milk quality and udder health in Ethiopia’s growing dairy sector. Future studies should incorporate molecular characterization of isolates and antimicrobial resistance profiling to monitor strain diversity and guide effective therapeutic interventions.

**Nomenclature**
CMT: California mastitis test
*S. aureus*
: *Staphylococcus aureus*
CI: Confidence interval
AOR: Adjusted odds ratio
RR: Right rear
LR: Left rear
RF: Right front
LF: Left front

## Author Contributions

Sossina Ashenafi Bijiju developed the study design, performed the laboratory work, and drafted the manuscript. Habtamu Tassew Tarekegne supervised the research work. Halo Yohannes Alemu participated in the data analysis and manuscript writing. Solomon Tibebu Melkie participated in the data analysis and manuscript writing. Sossina Ashenafi Bijiju, Halo Yohannes Alemu, Solomon Tibebu Melkie, and Habtamu Tassew Tarekegne critically reviewed the manuscript.

## Funding

This research work was part of a DVM thesis and was financially supported by Bahir Dar University, Ethiopia.

## Disclosure

A preprint version of this manuscript was previously posted on Research Square under the title “Prevalence of Bovine Mastitis and Isolation of *Staphylococcus aureus* from Cow Milk in Dessie Town, Northeast Ethiopia.” All authors read and approved the final manuscript.

## Ethics Statement

All the samples that included animals and humans were collected in accordance with the relevant ethical principles and guidelines. The animal study was conducted in accordance with ethical principles and guidelines for the use of animals for scientific purposes.

## Conflicts of Interest

The authors declare no conflicts of interest.

## Data Availability

The data that support the findings of this study are available from the corresponding author upon reasonable request.
